# The AI-based phase-seeding (AI-PhaSeed) method: early applications and statistical analysis

**DOI:** 10.1107/S1600576725008271

**Published:** 2025-10-18

**Authors:** Benedetta Carrozzini, Francesca Fedele, Anna Moliterni, Liberato De Caro, Corrado Cuocci, Cinzia Giannini, Rocco Caliandro, Angela Altomare

**Affiliations:** a Institute of Crystallography National Research Council via Amendola 122/o Bari 70126 Italy; Oak Ridge National Laboratory, USA

**Keywords:** crystal structure solution, phase seeding, artificial intelligence, AI phasing

## Abstract

The crystallographic challenge of structure determination is nowadays effectively supported by advanced computational methods, such as direct methods and Patterson techniques, implemented in sophisticated software. With the rapid expansion of artificial intelligence (AI) across diverse scientific domains, exploring its potential contribution to structure solution and its ability to overcome the limitations of traditional approaches has become increasingly compelling. This work builds upon and extends the findings of two recent studies on AI-driven phasing. The first, by Larsen *et al.* [*Science* (2024), **385**, 522–528], focused on designing and applying a neural network architecture to solve small structures (with unit-cell volumes up to 1000 Å^3^), primarily within the most common centrosymmetric space group *P*2_1_/*c*. The second, by Carrozzini *et al.* [*Acta Cryst.* (2025), A**81**, 188–201], introduced a novel phase-seeding method applicable to both centro­symmetric and non-centrosymmetric crystal structures of varying complexity, from small molecules to proteins. Although designed with AI integration in mind, this latter method had not yet been tested within an AI framework. In this paper, we apply the method proposed by Carrozzini *et al.* to cases where seed phases are generated by the AI network developed by Larsen *et al.* We demonstrate that this combined approach, termed AI-PhaSeed, successfully extends the applicability of Larsen’s neural network to structures with unit-cell volumes exceeding 1000 Å^3^, even under conditions of limited experimental resolution. The proposed procedure has been extensively tested on a set of structures taken from the Crystallography Open Database, proving it to be a powerful and reliable tool for structure solution. We also provide insights into the use of AI for crystallographic phasing and introduce statistical tools to evaluate the robustness of the solution process based on AI-calculated phases.

## Abstract 2

This paper highlights the significant contribution made by artificial intelligence to the phasing process, with a focus on its application to small structures ranging in volume from 1000 to 3500 Å^3^.

## 1. Abbreviations

AI: artificial intelligence.

DM: direct methods.

MPE: mean phase error.

*R*_f_: crystallographic agreement factor.

*N*_asym_: number of non-hydrogen atoms in the asymmetric unit.

*N*_refl_: number of measured symmetry-independent reflections.

EDM: electron-density modification.

*E*_*h*_: normalized structure factor for reflection *h*.

CORR: the correlation coefficient between phased and true electron-density maps.

## 2. Introduction

The phase problem in crystallography has long represented a central challenge, complicating the determination of crystal structures for organic, inorganic or metal–organic structures regardless of their asymmetric unit size, whether small (<80 non-hydrogen atoms), medium (<300) or large (≥300). Today, the routine solution of small- and medium-sized single-crystal structures using X-ray diffraction data collected in standard laboratories is largely feasible, provided the experimental data are of sufficient quality and resolution. This progress is the result of substantial theoretical and methodological advancements, including the development of direct methods (Giacovazzo, 2013 ▸), Patterson techniques (Rius, 2014 ▸) and dual-space approaches such as charge flipping (Oszlányi & Sütő, 2011 ▸). These approaches have been further empowered by significant improvements in diffractometer instrumentation and, crucially, in software packages that enhance modern computational capabilities (Burla *et al.*, 2015 ▸; Rius, 2011 ▸; Palatinus & Chapuis, 2007 ▸). Nevertheless, structure solution remains a challenge in some cases, especially for large mol­ecules. Difficulties also emerge when the experimental resolution is far from atomic, ∼1.2 Å according to the ‘Sheldrick rule’ (Sheldrick, 1990 ▸), which is the threshold at which individual atoms can be clearly resolved in an electron-density map.

The application of artificial intelligence (AI) is currently being explored across various scientific disciplines. In the field of structural crystallography, one of the most significant breakthroughs has been the development of *AlphaFold* by Google DeepMind (Jumper *et al.*, 2021 ▸), which has demonstrated unprecedented accuracy in predicting protein 3D structures directly from amino acid sequences. This milestone has underscored the transformative potential of AI in structural biology and opened new avenues for accelerating structure determination beyond traditional experimental approaches.

In a pioneering study, Larsen *et al.* (2024 ▸) demonstrated that AI can be effectively harnessed to address the phase problem in crystallography, successfully solving *ab initio* crystal structures with unit-cell volumes up to 1000 Å^3^ using only experimental amplitude data. Their deep learning architecture was specifically designed for small structures, primarily within the space group *P*2_1_/*c*, and was trained on a dataset comprising millions of artificial crystal structures. While the work of Larsen *et al.* (2024 ▸) was focused on a relatively narrow class of structures, some of which may be solvable through traditional direct methods or Patterson techniques, its findings represent a major step forward. Notably, the study demonstrated that AI can determine missing phases, a task that has traditionally relied on decades of theoretical and algorithmic development in crystallography. Beyond its immediate achievements, this work opened new avenues for applying AI to more complex scenarios that remain challenging for conventional methods, particularly when the experimental resolution is limited. These opportunities can be further explored using the recently developed phase-seeding method (Carrozzini *et al.*, 2025 ▸), which was explicitly designed to be AI compatible and applicable to crystal structures of any size and space group. The method is based on the accurate identification of a small subset of seed phases; when these are combined with experimentally derived amplitudes and randomly initialized phases, they can drive the reconstruction of the full electron-density map. This is accomplished by iterative procedures in both direct and reciprocal space, enhanced by electron-density modification (EDM) cycles to extend and refine the phase set. For non-centrosymmetric structures, the method introduces a discretization of both seed and random phases into a limited number of distinct values, thereby enabling classification and refinement through an AI-driven algorithm. Although this method has been robustly defined and tested *in silico*, it had not yet been implemented in a fully AI-based framework prior to the present work.

This paper presents the first application of the phase-seeding method, in combination with the neural network developed by Larsen *et al.* (2024 ▸), extending its use to structures still within the space group *P*2_1_/*c* but with unit-cell volumes ranging from 1000 Å^3^ up to 3500 Å^3^, well beyond the previously studied limit below 1000 Å^3^. These structures include organic, inorganic and metal–organic compounds. Despite the original design constraints of the neural network, the combined AI-based phase-seeding approach has demonstrated promising performance, benefiting from the advances introduced by the phase-seeding method, which requires reliable phase estimates for only a limited subset of reflections.

The novelty of this study lies in its clear demonstration that the AI model developed by Larsen *et al.* (2024 ▸) can be extended beyond its initial volumetric limitations through the integration of the phase-seeding strategy. We provide a comprehensive assessment of the capabilities and limitations of the AI-based phase-seeding (AI-PhaSeed) method. Validated on a large dataset of experimentally determined structures, AI-PhaSeed proves to be a robust and competitive alternative to traditional phasing approaches such as direct methods (DM).

Particularly noteworthy is the section addressing phasing from limited-resolution data, where we demonstrate that AI-PhaSeed can successfully solve structures that remain intractable using traditional methods.

## 3. Methods

### 3.1. The AI-based phase-seeding (AI-PhaSeed) method

The AI-based phase-seeding method integrates the concept of phase seeding with AI to solve crystal structures from X-ray diffraction data, once the unit cell and space group have been determined. Its effectiveness has been tested on real small-sized structures with unit-cell volumes ranging from 1000 to 3500 Å^3^ and space group *P*2_1_/*c*, deposited in the Crystallography Open Database (COD) (Gražulis *et al.*, 2009 ▸; Downs & Hall-Wallace, 2003 ▸).

**Figure 1:**
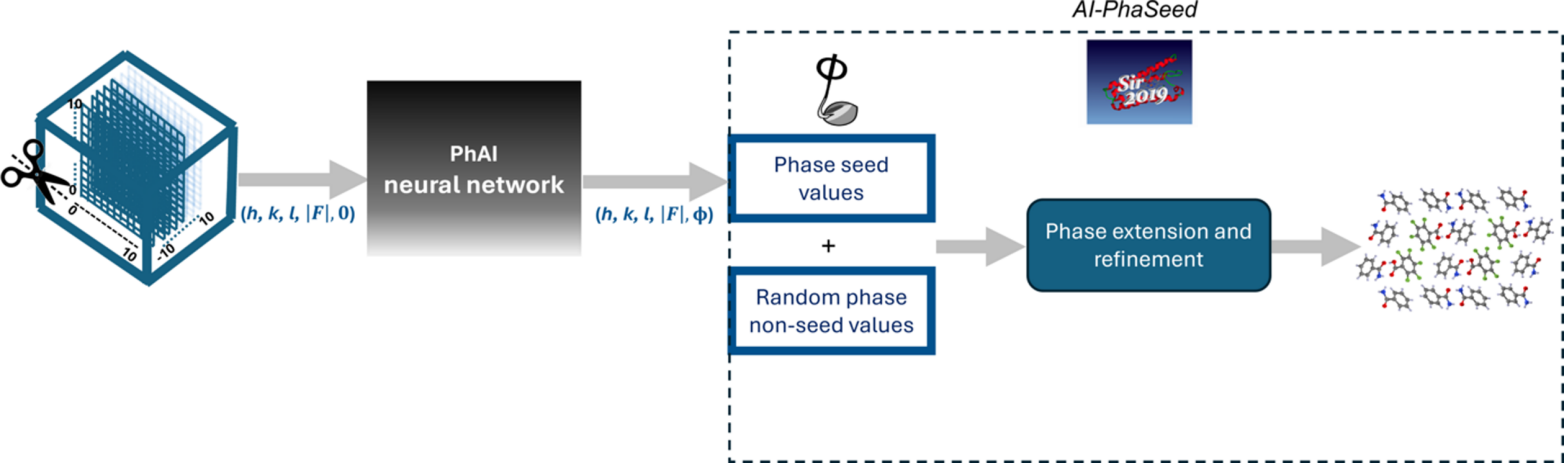
Scheme of the AI-PhaSeed method. The image of the phase seeding is taken from Madsen (2025 ▸) and reproduced with permission.

AI-PhaSeed involves the following steps outlined in Fig. 1 ▸:

(i) For each real structure in the centrosymmetric space group *P*2_1_/*c*, a subset of experimental reflections is selected to meet the input size requirements of the PhAI neural network (Larsen *et al.*, 2024 ▸). The selected reflections follow the constraint on Miller indices (*hkl*) such that (*h*^2^ + *k*^2^ + *l*^2^)^1/2^ ≤ 10 and, due to the Laue symmetry 2/*m*, −10 < *h* < 10, 0 < *k* < 10 and 0 < *l* < 10. This selection is performed automatically by a built-in tool within *SIR2024*, the latest updated version of the *SIR2014* package (Burla *et al.*, 2015 ▸). For each *hkl* reflection, the amplitude and an initial phase set to zero are provided as input to PhAI, and the run was set with five phasing cycles.

This subset constitutes only a small fraction of the total number of reflections for each structure.

(ii) The phases predicted by the PhAI network (restricted to 0 or π) provide the phase-seed values required by the phase-seeding procedure (Carrozzini *et al.*, 2025 ▸). The efficiency of the AI-generated phases is evaluated using the mean phase error (MPE_seed_), calculated as the average difference between the AI-derived phases and those derived from the known structural model, as well as the correlation coefficient (CORR_seed_) as defined by Larsen *et al.* (2024 ▸).

(iii) For non-seed reflections, phases are randomly assigned as either 0 or π. The subsequent phase extension and refinement procedure employs iterative EDM cycles, using the experimental amplitudes as constraints (Carrozzini *et al.*, 2025 ▸). The final electron-density map is computed during this process, which is executed using the *SIR2024* software (Burla *et al.*, 2015 ▸). Three validation parameters are used to evaluate the reliability and accuracy of both the phasing process and the resulting structure solution: (1) the final mean phase error (MPE_final_), calculated in reciprocal space as the deviation between the refined and model-derived phases; (2) the final correlation coefficient (CORR_final_); and (3) the crystallographic agreement factor (*R*_f_) obtained by comparing calculated and observed amplitudes.

### 3.2. AI-PhaSeed combined with direct methods

**Figure 2:**
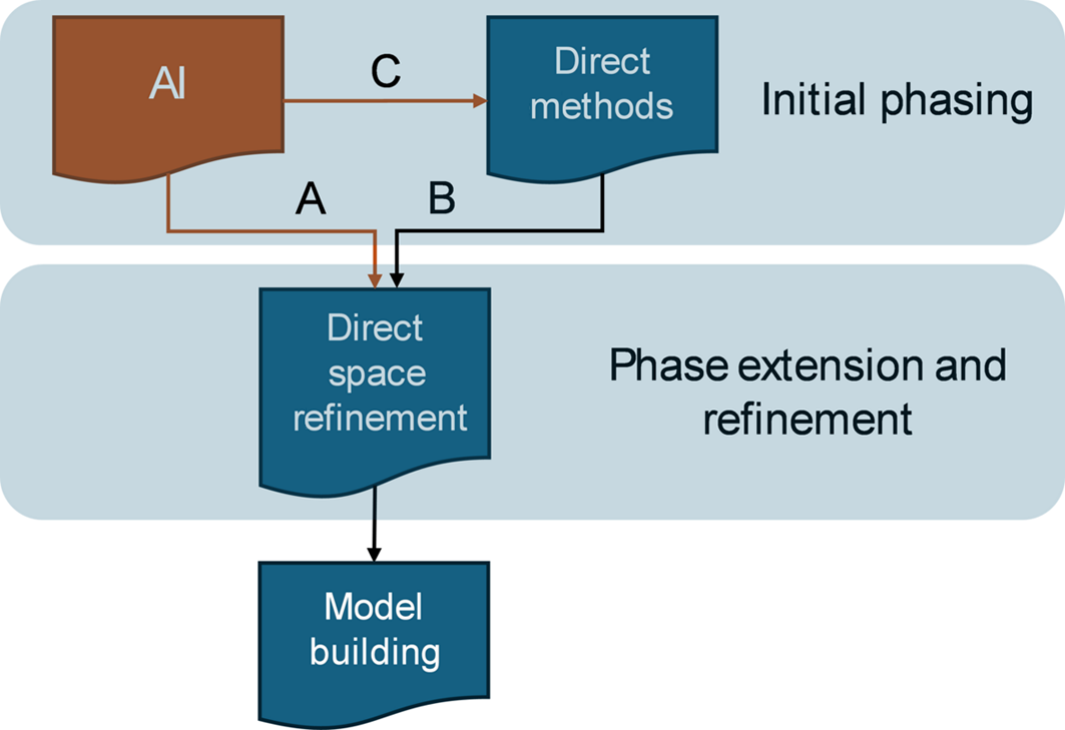
Workflow of the AI-PhaSeed method (path A), the classical phasing procedure (path B) and their combination DM+AI-PhaSeed (path C).

In the AI-PhaSeed method, the phases generated by AI are extended and refined using direct-space methods applying cycles of electron-density map modifications (Burla *et al.*, 2010 ▸) (path A in Fig. 2 ▸). The conventional phasing protocol, typically performed using DM for small- and medium-sized compounds, involves initial phase determination followed by phase extension and refinement in direct space, the same approach as adopted in the AI-PhaSeed method (path B in Fig. 2 ▸).

In this study we present the first applications of the AI-PhaSeed method and compare its performance with the classical DM phasing procedure (path A versus path B in Fig. 2 ▸). In addition, we propose a strategy, called DM+AI-PhaSeed (see Section 4.2), that combines the two procedures (path C in Fig. 2 ▸), based on the theory outlined in Section 3.3. In this approach, the AI-generated phase seed is actively integrated into DM to drive the multi-solution process more effectively towards a reliable phase set.

### 3.3. AI-based tangent formula

The direct methods approach exploits structure invariants, phase combinations unaffected by origin shifts, typically derived from a set of strong reflections (|*E*_*h*_| > 1.2), referred to as *N*_large_. Phases are estimated from known amplitudes via the tangent formula (Karle & Hauptman, 1956 ▸), which provides probabilistic phase values based on statistical relationships. The phasing process starts with random phase assignment to *N*_large_ reflections followed by iterative refinement. Since convergence is not always achieved, the procedure is repeated with different random inputs, a multi-solution approach that increases the chance of identifying correct phase estimates.

According to the formalism described by Giacovazzo (1998 ▸, pp. 123–127), the total probability *P*(θ_*h*_) that the phase of *E*_*h*_ involved in *r* structure-invariant relationships (specifically triplets, in this case) equals θ_*h*_ can be expressed as a suitably normalized product of *r* specific probabilities,
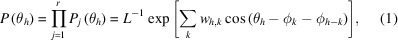
where *L* is a normalizing factor, and ϕ_*k*_ and ϕ_*h*−*k*_ are phases of reflections contributing to the invariants. Usually, for the tangent formula, we have
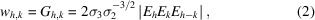
where 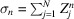 and *Z*_*j*_ is the atomic number of the *j*th atom.

When *a priori* phase information is available, such as AI-derived phase estimates for a subset of *N*_large_ reflections, this information can be directly combined into equation (1), with random phases assigned to the rest. *A priori* phase information could be generated by running a neural network multiple times with different random seeds. However, results from this study indicate that performing multiple random trials to generate phase histograms offers limited benefit. Consequently, a single AI-derived phase seed, as used in this work, is sufficient to drive the phasing process effectively, reducing computational cost without compromising accuracy.

Thus, incorporating *a priori* knowledge from AI-derived phase values with reliability 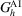, the total probability *P*_tot_(θ_*h*_) that the phase of *E*_*h*_ is θ_*h*_ can be written as (Giacovazzo, 1998 ▸, pp. 564–565)
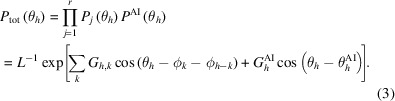


If we treat the AI-derived phase estimates as triplets, we can always estimate 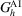 through equation (2) and we have
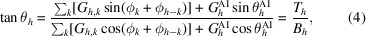
with statistical weight given by
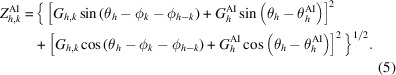


Equations (3)–(5) generalize the tangent formula to account for *a priori* knowledge from AI-derived phase estimates. When AI estimates of both ϕ_*k*_ and ϕ_*h*−*k*_ are available, they jointly contribute to equation (5), enhancing the reliability of the corresponding structure invariant. If only one phase is available, it still provides a partial but meaningful enhancement to the triplet’s reliability. This approach allows external information (AI estimates) to be incorporated in a natural and consistent manner within the formalism of DM. The *a priori* phase information does not override the estimates derived from triplet relationships, but rather merges with them according to their relative reliability. Mathematically, this is equivalent to the weighted vector sum of phase estimates in the complex plane.

### 3.4. Test structures and relevant variables

Test structures were obtained from the COD (Gražulis *et al.*, 2009 ▸; Downs & Hall-Wallace, 2003 ▸) using tools integrated into the software package *EXPO* (Altomare *et al.*, 2013 ▸). We selected 1505 crystal structures in the *P*2_1_/*c* space group, all solved using single-crystal X-ray diffraction data. This choice aligns with the PhAI neural network (Larsen *et al.*, 2024 ▸) used in our study to generate AI phases, which was specifically trained on structures in the *P*2_1_/*c* space group. In the AI-PhaSeed method, the AI-generated phases are subsequently extended and refined. Additionally, in accordance with the capabilities of the PhAI neural network designed to perform phasing on datasets with unit-cell volumes below 1000 Å^3^ and constrained Miller indices (*hkl*), we restricted our selection to structures with unit-cell volumes between 1000 and 3500 Å^3^. This range ensures compatibility with the AI-PhaSeed method, which, according to Carrozzini *et al.* (2025 ▸), requires phase estimates for a subset (typically 10%) of the total reflections. The main features of the selected structures are summarized in Table 1 ▸, and they are also illustrated as box plots in Fig. S1 in the supporting information.

## 4. Results

As a first check, we verified the performance of the standalone PhAI method (without phase seeding or EDM cycles) on the dataset used in this study. The results, shown in Fig. S2, indicate that, even for structures for which the phase values assigned by PhAI are reliable (MPE_seed_ < 20°), the low-resolution electron-density map that can be calculated using them cannot be properly interpreted in terms of an atomic model. As a result, the percentage of correctly positioned atoms is less than 20% for most of the test structures. Thus, adopting the phase-seeding strategy to generate the PhAI phases is essential for the structures considered in this study.

**Figure 3:**
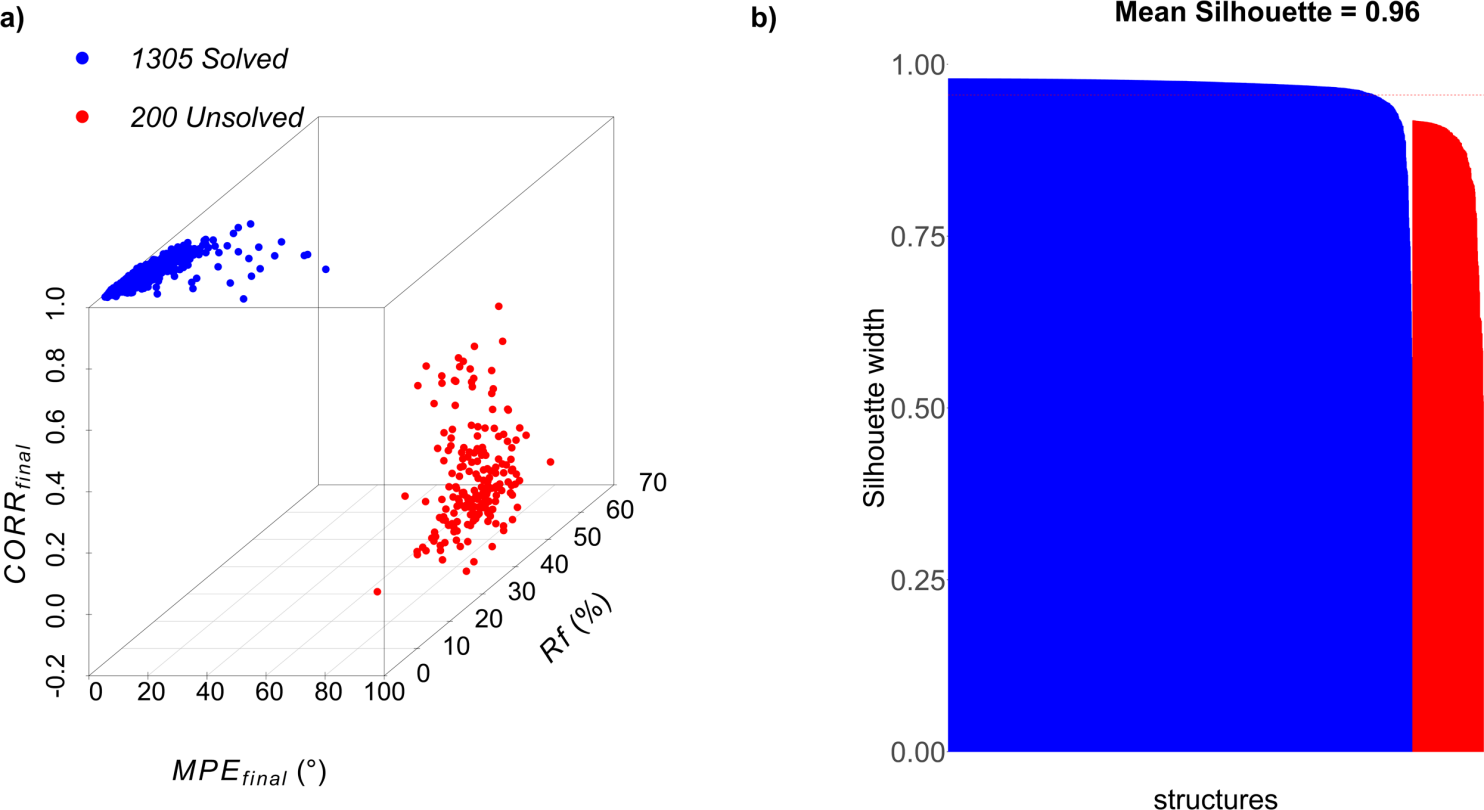
(*a*) A 3D scatter plot illustrating the *k*-means clustering results, with *k* = 2 (Hartigan–Wong algorithm), based on MPE_final_ (*x* axis), *R*_f_ (*y* axis) and CORR_final_ (*z* axis). Data points are colour-coded to distinguish between ‘Solved structures’ (blue) and ‘Unsolved structures’ (red), with the legend indicating the number of observations in each category. (*b*) A silhouette plot for *k*-means clustering with *k* = 2. Each block contains a number of vertical bars corresponding to the elements assigned to that cluster (blue for solved and red for unsolved structures). The height of each bar represents the silhouette width, indicating how well each element fits within its assigned cluster compared with the other one. Silhouette values closer to 1 correspond to better-defined clustering. The value of the mean silhouette width, shown above the plot, quantifies the overall clustering quality; values above 0.7 are generally considered indicative of strong and well separated clusters (Kaufman & Rousseeuw, 1990 ▸).

Given the size of the test dataset (1505 structures), the performance of the AI-PhaSeed method must be evaluated by a robust metric capable of automatically and unambiguously distinguishing solved structures from unsolved ones. To this end, we considered the space defined by three validation parameters (MPE_final_, CORR_final_ and *R*_f_), each calculated with respect to the published structure, to evaluate the outcome of the structure solution process. Representative points of each test structure are projected into this space [Fig. 3 ▸(*a*)] and clustered by a *k*-means clustering procedure with *k* = 2 (Hartigan & Wong, 1979 ▸). This results in two identified clusters: one representing the solved structures [blue points in Fig. 3 ▸(*a*)] and the other representing the unsolved structures [red points in Fig. 3 ▸(*a*)]. The two clusters are clearly separated, with the points corresponding to the solved structures accumulating around the optimal values of the validation parameters. As a result, the silhouette plot shown in Fig. 3 ▸(*b*) is dominated by the cluster of solved structures, most of which have a silhouette width larger than 0.8.

**Figure 4:**
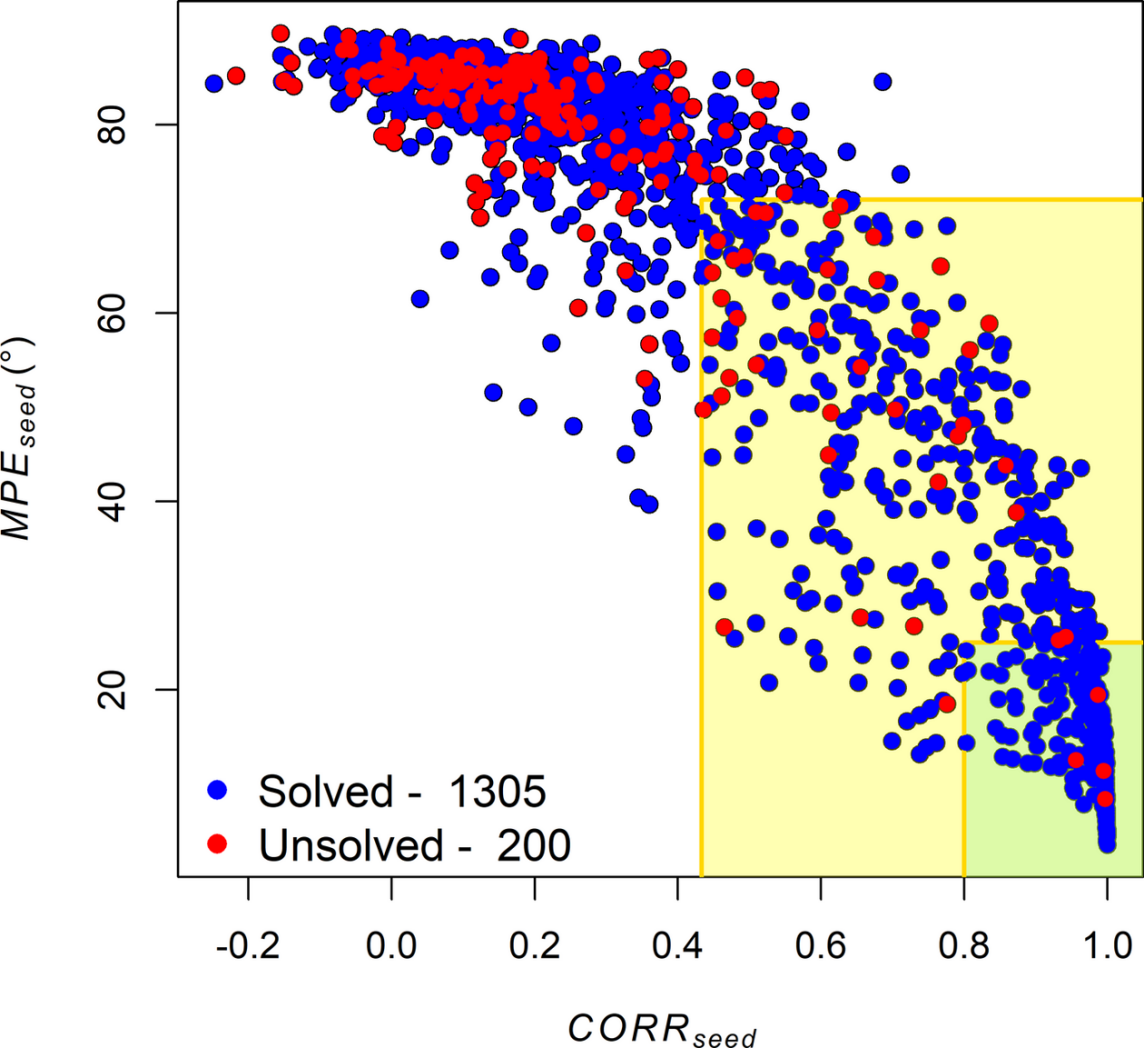
Scatter plot of MPE_seed_ versus CORR_seed_, showing clusters of solved (blue dots) and unsolved (red dots) structures. The green region corresponds to the area defined by MPE_seed_ ≤ 25 and CORR_seed_ ≥ 0.8 where the AI-generated phases can be considered reliable, *i.e.* sufficiently accurate to approximate the true phases. The yellow region corresponds to the area defined by optimizing the values MPE_seed_ ≤ Q1 and CORR_seed_ ≥ Q3 (Class 1). This region predominantly contains solved structures, which tend to accumulate at CORR_seed_ values close to 1.

The unambiguous definition of the solved structures allows us to calculate the efficiency of the AI-PhaSeed method as the ratio of solved to total structures, which comes to 86.7%. This value should be compared with that obtained using a random phase seed (random phases replacing AI phases), which is 84.3%, and that from a true phase seed (phases calculated from published structures replacing AI phases), which is 97.1%. As expected, the AI-PhaSeed method performs slightly better than the random-phase case but is quite far from what can be obtained using known phases. This gap is mainly due to a subset of structures for which AI fails to assign reliable phase values to seed reflections. However, if we focus on structures whose AI-generated seed phases exhibit a mean phase error (MPE_seed_) below 25° and a reflection correlation (CORR_seed_) greater than 0.8, *i.e.* the 300 structures for which the AI predictions can be considered reliable, the efficiency of the AI-PhaSeed method reaches 98%, closely aligning with the 97% achieved by the true-phase method on the same subset of structures. This is better shown in Fig. 4 ▸, where the *k*-means clusters of solved and unsolved structures are projected in the space defined by the two variables MPE_seed_ and CORR_seed_ (see Section 3.1): the green box contains the 300 structures with MPE_seed_ below 25° and CORR_seed_ greater than 0.8, and only four of them (red dots in the green box) belong to the unsolved *k*-means cluster. This outcome suggests that the AI-PhaSeed method performs as proposed by Carrozzini *et al.* (2025 ▸), assuming the AI-predicted seed phases are reliable.

However, this high efficiency is achieved for only approximately 20% of the total structures, specifically those that closely match the features of the neural network’s training set. To investigate the nature of the decline in AI efficiency and identify the structures for which this occurs, we developed a classification model to explain the AI-PhaSeed results.

To this end, we focused on analysing the intermediate result of the method, the one obtained after application of AI and related to the seed phases, by considering the two variables MPE_seed_ and CORR_seed_.

Well separated distributions, with a minimal overlap, are obtained when comparing the MPE_seed_ and CORR_seed_ values for structures belonging to the solved *k*-means cluster with those belonging to the unsolved *k*-means cluster. This result is illustrated in Fig. S3. The observed separation, while confirming the discriminative power of MPE_seed_ and CORR_seed_ in distinguishing between solved and unsolved structures and thus confirming the impact of AI-generated phases on the performance of the AI-PhaSeed method, also allows the identification of a threshold value for both variables, *i.e.* the first quartile (Q1) for MPE_seed_ and the third quartile (Q3) for CORR_seed_, both of the unsolved cluster. This ensures the identification of the maximum number of solved structures, while simultaneously minimizing the number of unsolved ones. These thresholds identify the yellow box in Fig. 4 ▸, which mostly contains solved structures accumulating at CORR_seed_ values close to 1.

We employed a random forest (RF) classification model (Breiman, 2001 ▸) as a supervised learning algorithm, labelling structures within the yellow region in Fig. 4 ▸ as Class 1 and all others as Class 0. The model was thus trained to classify as successful (Class 1) those structures with MPE_seed_ ≤ Q1 and CORR_seed_ ≥ Q3, *i.e.* those falling within the yellow box in Fig. 4 ▸, and as unsuccessful (Class 0) those lying outside this region. The main goal in building the classification model is to study how the quality of the AI phases influences the outcome of the structure solution. Consequently, a small number of structures (approximately 3% of the overall dataset) that remain unsolved even with the true phase seed are excluded from this stage of the analysis to avoid biases unrelated to phase quality.

**Figure 5:**
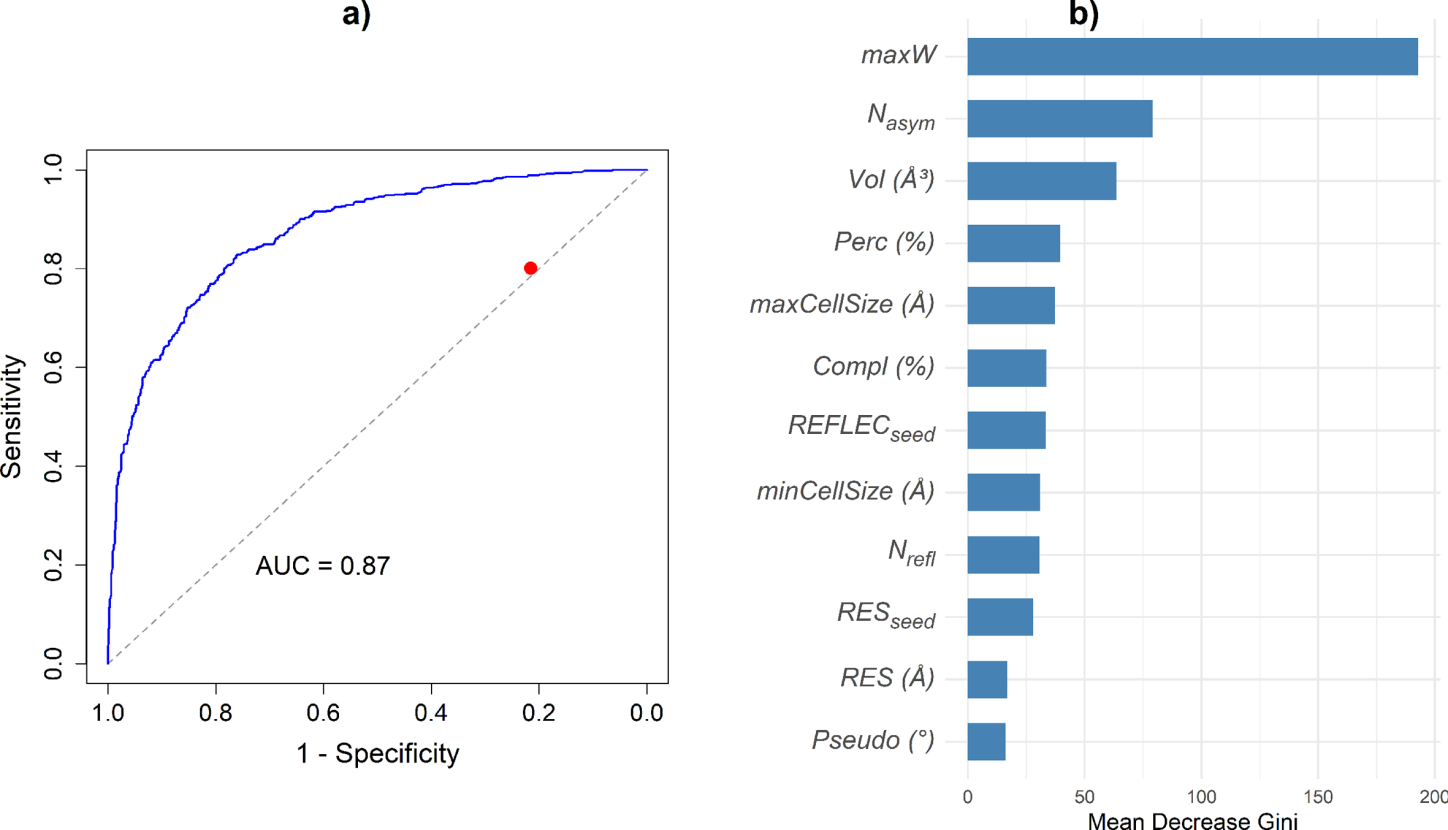
(*a*) The ROC curve of the RF model, illustrating sensitivity and specificity values across varying thresholds. The red dot marks the optimal cut-off point, identified as the highest specificity value corresponding to a specificity of at least 80%, for the correct classification of a significant portion of Class 1 structures. The AUC is reported as a measure of the overall performance of the model. (*b*) Feature importance, ordered by the descending mean decrease Gini (Kaufman & Rousseeuw, 1990 ▸) resulting from the RF model.

The RF results are shown in Fig. 5 ▸. The optimal probability threshold on the receiver operating characteristic (ROC) curve (Fawcett, 2006 ▸) [red dot in Fig. 5 ▸(*a*)] was determined to classify correctly a significant portion of Class 1 structures by selecting the highest sensitivity value corresponding to a specificity of at least 80%. The area under the curve (AUC) value is 0.87, indicating an excellent overall model performance. The resulting confusion matrix for the tenfold cross-validated RF model is reported in Table 2 ▸.

The RF model can be effectively used to optimize the decision-making process and resource allocation in crystal structure determination by applying the AI-PhaSeed method only to structures classified as Class 1. For these structures, the efficiency of AI-PhaSeed increases to 90%, compared with 85% with a random phase seed. This indicates that when AI provides reliable seed phases the AI-PhaSeed method performs close to the optimal scenario where true phases are used as seeds (96% efficiency).

As noted above, a key advantage of developing a classification model to assess the performance of AI-PhaSeed is its ability to evaluate the importance of different structural variables in determining the success of the phasing process. The feature importance, ordered by the descending mean decrease Gini coefficient (Kaufman & Rousseeuw, 1990 ▸), is shown in Fig. 5 ▸(*b*) and reveals that max*W*, *i.e.* the largest atomic weight among the elements in the unit cell, is a key variable. This reflects a well established principle in crystallography: the presence of heavy atoms can facilitate the phasing process. What is particularly noteworthy is that the importance of this feature was identified by the RF classification model, even though no explicit information about atomic weight was provided during the neural network’s training phase. This represents a significant insight into the application of AI in structure determination, as it highlights the deep learning model’s capacity to extract physically meaningful information from data autonomously.

**Figure 6:**
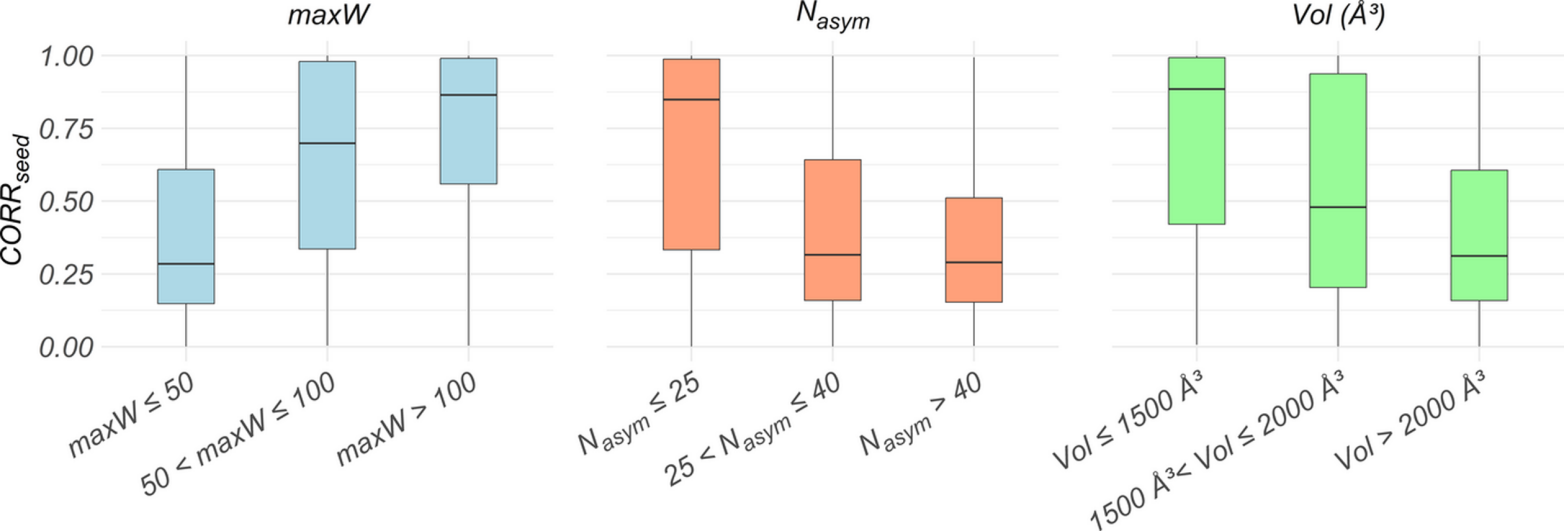
Distribution of CORR_seed_ across different intervals of the three most important features according to the RF mean decrease Gini [Fig. 5 ▸(*b*)]: maximum atomic weight among the structure elements (max*W* ≤ 50, 50 < max*W* ≤ 100 and max*W* > 100), number of non-H atoms in the asymmetric unit (*N*_asym_ ≤ 25, 25 < *N*_asym_ ≤ 40 and *N*_asym_ > 40) and unit-cell volume (Vol ≤ 1500 Å^3^, 1500 < Vol ≤ 2000 Å^3^ and Vol > 2000 Å^3^). Horizontal black bars indicate median values.

Subsequent relevant variables, ordered by the mean decrease Gini (Kaufman & Rousseeuw, 1990 ▸) in Fig. 5 ▸(*b*), are *N*_asym_, Vol and maxCellSize, related to the size of the unit cell in direct space, and Perc and REFLEC_seed_, related to the size of the unit cell in reciprocal space. These findings indicate that another key parameter influencing the efficiency of the AI-based phase assignment is the complexity of the crystal structure (*e.g.* in terms of number of non-H atoms in the asymmetric unit). This is closely linked to the limited size of the phase seed: for a larger crystal structure more effort is required to extend the phase information to all measured reflections. To verify the effect of the variables highlighted by the feature importance analysis, we plot in Fig. 6 ▸ the distribution of CORR_seed_ across different intervals of the three most important features according to the RF mean decrease Gini [Fig. 5 ▸(*b*)]: max*W* (max*W* ≤ 50, 50 < max*W* ≤ 100 and max*W* > 100), *N*_asym_ (*N*_asym_ ≤ 25, 25 < *N*_asym_ ≤ 40 and *N*_asym_ > 40) and Vol (Vol ≤ 1500, 1500 < Vol ≤ 2000 and Vol > 2000). As expected, a clear increase in CORR_seed_ values is observed in intervals closer to the optimal values of the variable, *i.e.* for higher max*W* values and lower *N*_asym_ and Vol values.

As a result of the classification method, we can now estimate the performance on Class 1 structures. The AI-PhaSeed method shows improved efficiency, increasing from 86.7% on the entire dataset to 91.1% when applied specifically to the Class 1 subset.

### 4.1. AI phasing at limited data resolution

Given the challenging task of solving structures at resolutions far from the atomic one, we explored the performance of AI-PhaSeed as the data resolution was progressively decreased, being aware that the size of the seed relative to the total number of observed reflections plays a significant role. The analysis revealed that both MPE_seed_ and CORR_seed_ values are distributed differently across the datasets obtained after applying the resolution cut-offs (*i.e.* 1.0, 1.2, 1.4 and 1.6 Å). Additionally, the criteria for distinguishing solved from unsolved structures and for separating Class 0 and Class 1 structures after application of AI become less distinct compared with the corresponding results without a resolution cut-off. However, the mean silhouette width value remains above 0.7 (Kaufman & Rousseeuw, 1990 ▸), still indicating an appreciable degree of separation. Figs. S4, S5 and S6 present the statistical analysis of the results obtained using a 1.6 Å resolution cut-off, following the same procedure as the tests on native uncut structures. At this resolution, 23 structures exhibited statistical parameters that were unsuitable for applying DM (*e.g.* an insufficient number of structure-invariant relationships) and were therefore excluded from the dataset. The results of the RF model (Fig. S7 and Table S1) and the validation of the feature importance analysis (Fig. S8) are also provided.

The performance of the RF model is characterized by a still acceptable AUC of 0.75. The variable Perc shows increased importance compared with its role in the model trained on uncut data, emerging as the second most important variable after max*W*, as shown in Fig. S7(*b*) [compare with Fig. 5 ▸(*b*)].

**Figure 7:**
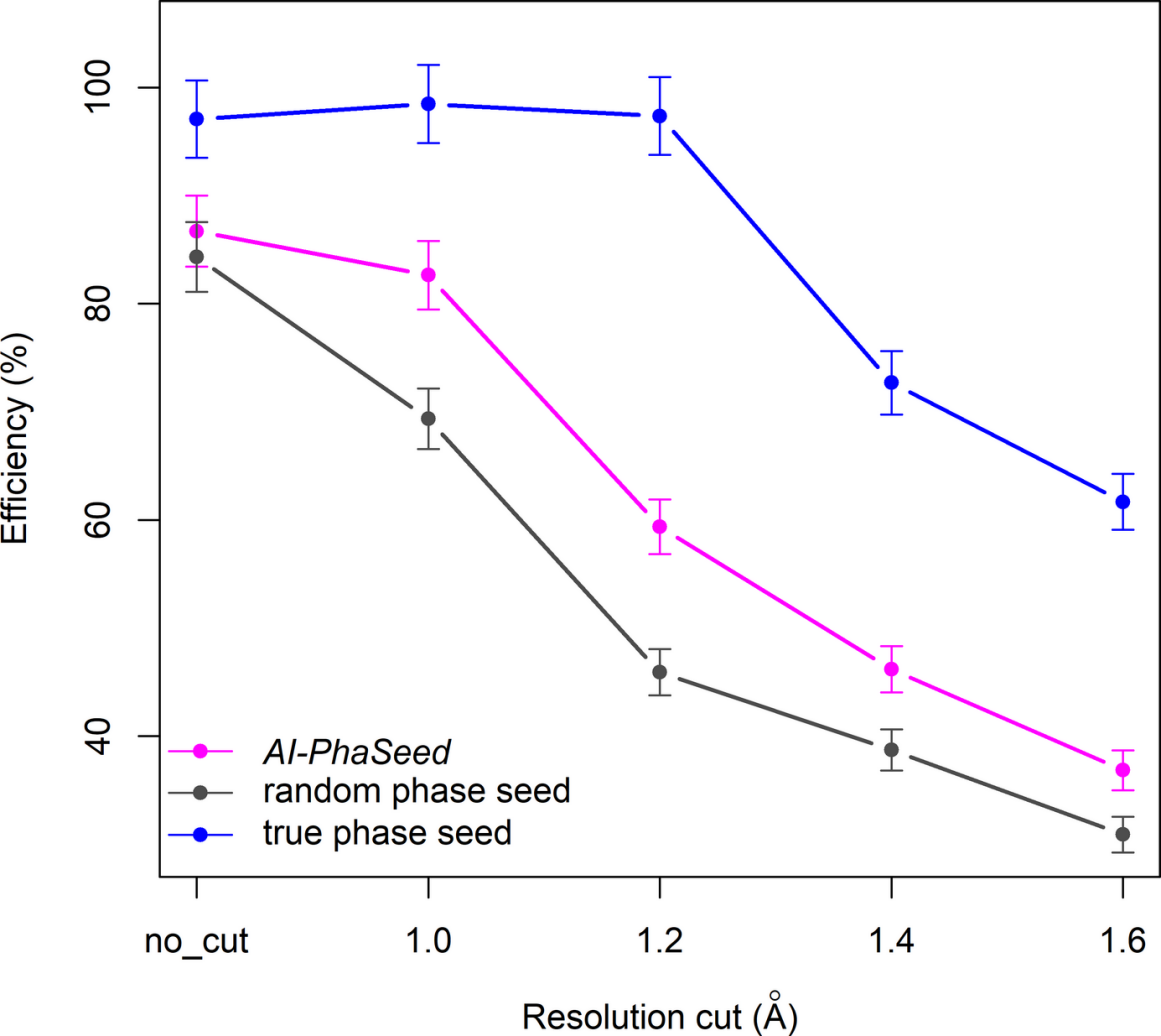
Efficiency of the AI-PhaSeed method as a function of data resolution cut-offs, compared with the efficiency obtained using random phase and true phase seeds. Five datasets were analysed: no resolution cut (no_cut), and cut-offs at 1.0, 1.2, 1.4 and 1.6 Å resolution. Error bars represent the propagated uncertainty, calculated as 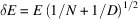, where *E* is the efficiency (%), *D* is the number of correctly classified structures and *N* is the total number of structures.

Similar evaluations have been performed for all datasets corresponding to the various resolution cut-offs. The trend in AI-PhaSeed efficiency (*E*) plotted against the resolution cut-off is shown in Fig. 7 ▸ and compared with that obtained by feeding the seed with random or true phase values. In Fig. 7 ▸, error bars represent the propagated uncertainty, calculated as 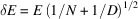, where *E* is the efficiency (%), *D* is the number of correctly classified structures and *N* is the total number of structures. A common decreasing trend is observed across the curves, although the rates of decline differ. In particular, it can be noted that the efficiency of the true phase seed remains unaffected by the resolution cut-off up to 1.2 Å, while it drops substantially at 1.4 Å. Conversely, the random phase seed has a steady decrease in efficiency for data resolution up to 1.2 Å, which becomes less steep when going to 1.4 Å data resolution. The efficiency of the AI-PhaSeed method is always intermediate between the random and true phase seed. The decrease in the rate of efficiency follows that of the random-phase-seed curve for data resolution between 1.0 and 1.2 Å, while it is less steep for data resolution <1.0 Å or between 1.2 and 1.4 Å.

**Figure 8:**
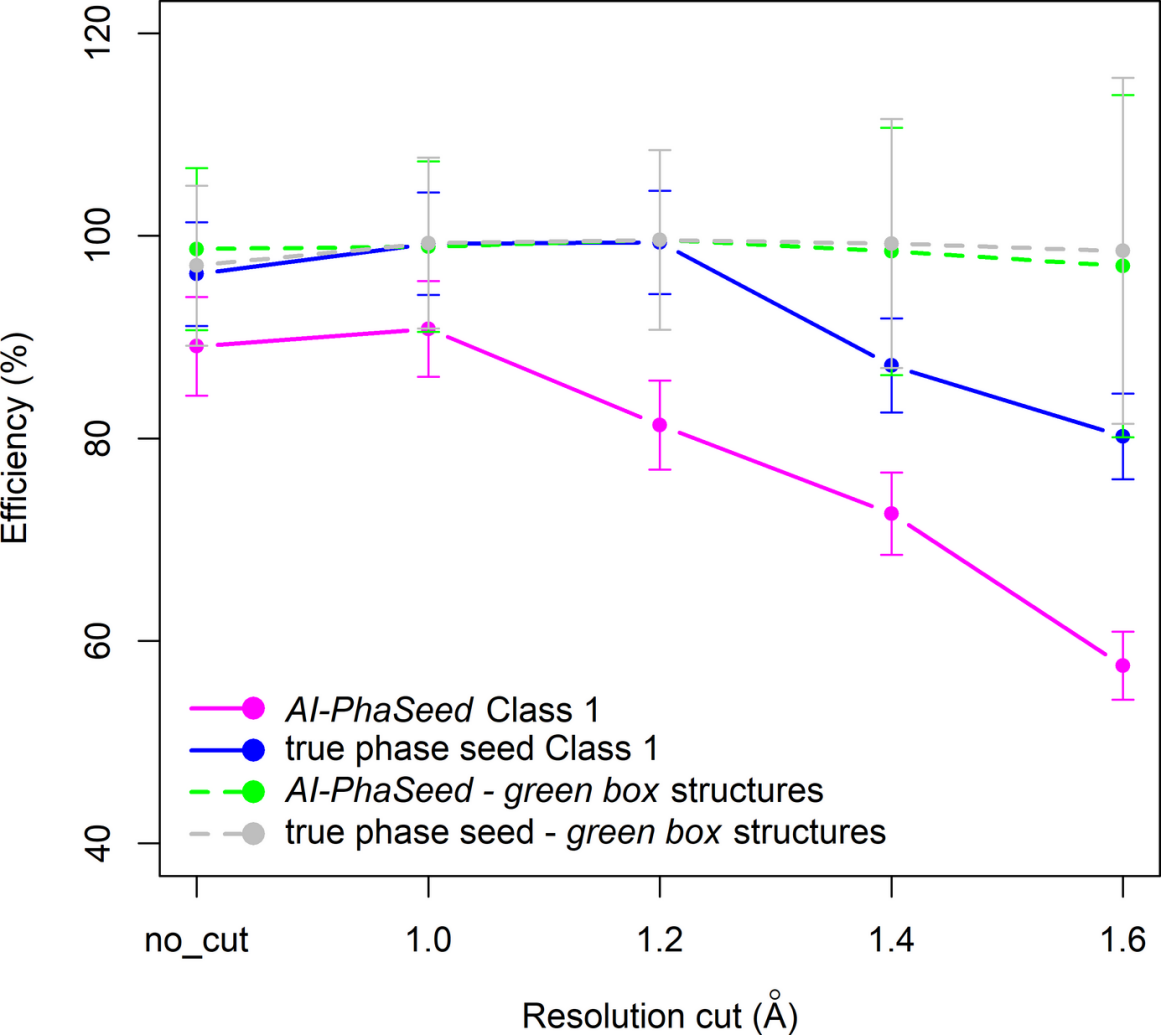
Efficiency of the AI-PhaSeed method applied on subsets of test structures as a function of the data resolution cut-offs, compared with the efficiency obtained using true phases (true phase seed). Full and dashed lines represent the efficiency calculated for structures belonging to their corresponding resolution-cut-off-based yellow (Class 1) and green boxes, as reported in Fig. S6. Error bars are also shown.

An RF classification model was also built for each resolution cut-off, following the same procedure described above. As shown in Fig. 8 ▸, an increase in the efficiency of the AI-PhaSeed method for Class 1 structures with respect to the full set of structures (Fig. 7 ▸) is observed across all datasets, resulting in a smaller gap with the true phase-seeding efficiency. A satisfactory efficiency, close to 60%, for Class 1 structures is reached even at 1.6 Å resolution. Fig. 8 ▸ also shows the AI-PhaSeed efficiency calculated for structures with AI-generated phases very close to the true phases (MPE_seed_ < 25 and CORR_seed_ > 0.8), located in their corresponding green boxes as shown in Fig. S6 for the case at 1.6 Å; the efficiency is very close to 100%, regardless of the resolution cut-off, and almost coincides with the true phase-seeding values.

### 4.2. AI-PhaSeed combined with DM

**Figure 9:**
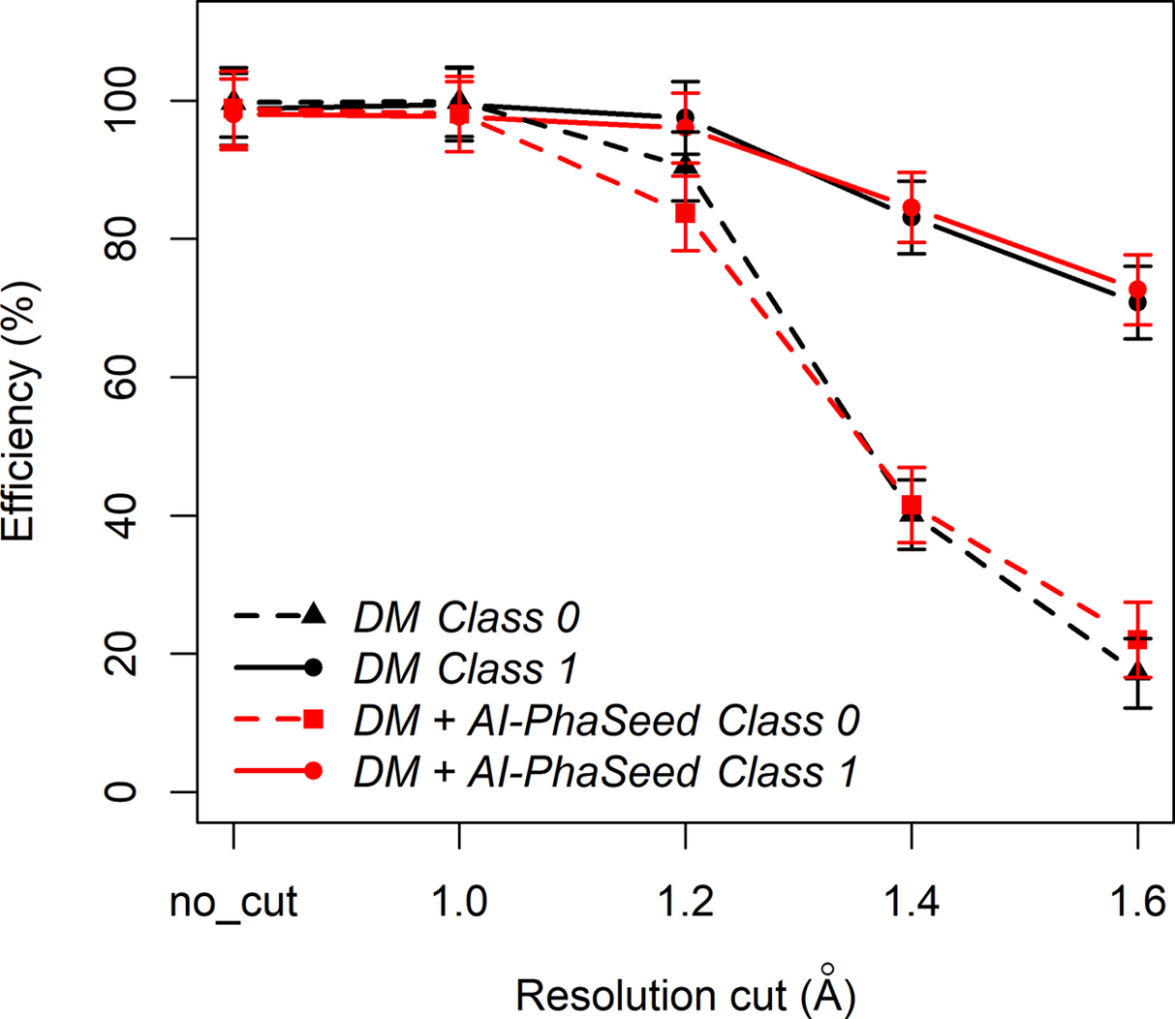
Efficiency of the DM and DM+AI-PhaSeed methods when applied to Class 0 and Class 1 test structures, plotted as a function of applied resolution cut-off level. Five different datasets were compared: no resolution cut (no_cut) and cut-offs at 1.0, 1.2, 1.4 and 1.6 Å resolution (Class 1 corresponds to MPE_seed_ ≤ Q1 and CORR_seed_ ≥ Q3, and Class 0 to MPE_seed_ > Q1 and CORR_seed_ < Q3). Error bars are also shown.

**Figure 10:**
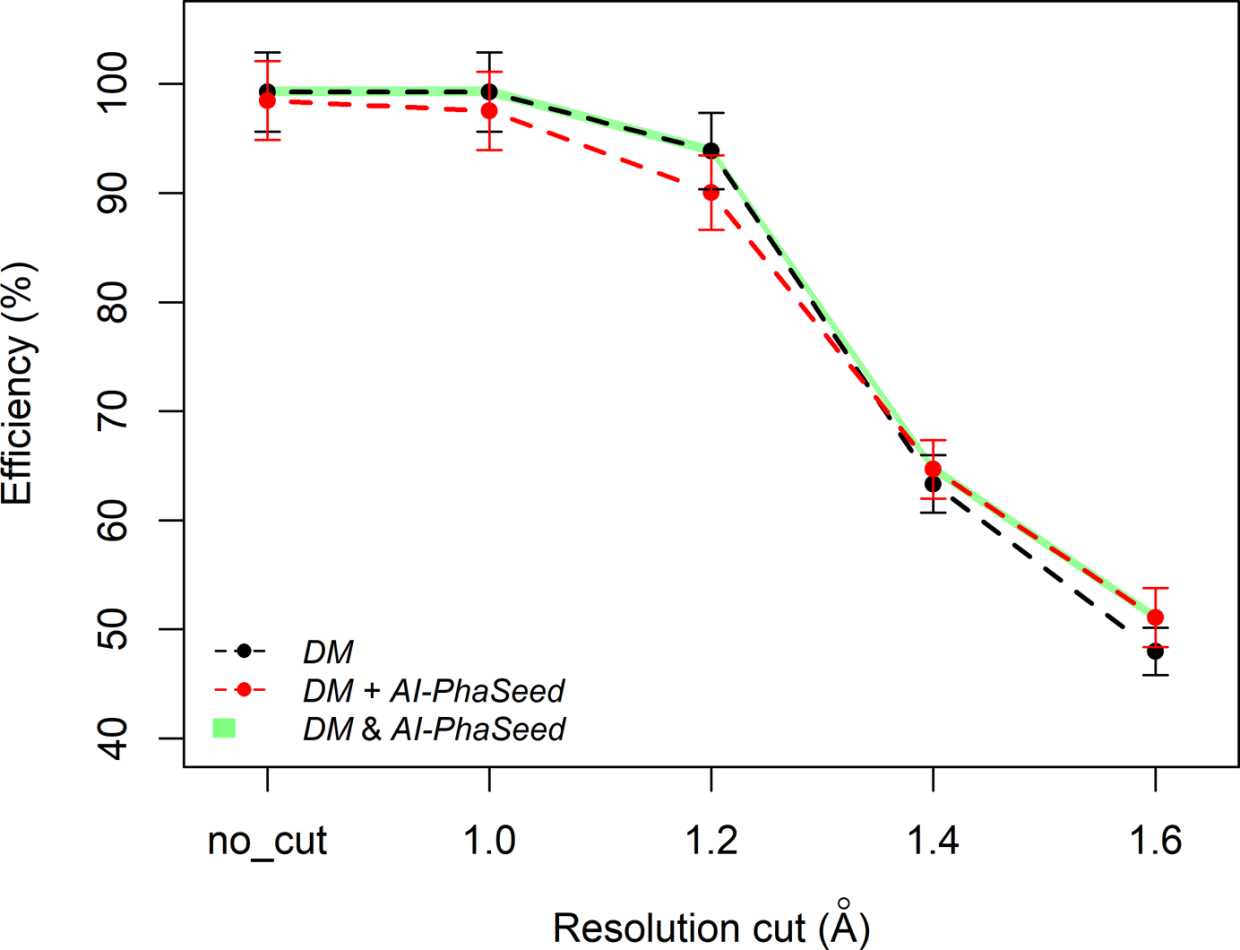
Efficiency of the AI-PhaSeed method combined with DM, plotted as a function of resolution cut-off level, compared with that obtained using DM alone and with the protocol DM&AI-PhaSeed.

The newly introduced AI-PhaSeed needs to be compared with the gold standard for *ab initio* crystal structure solution of small molecules, *i.e.* DM. With an efficiency of 86.7% on the entire dataset, AI-PhaSeed performs significantly below DM, which achieves 99.1% efficiency. This difference is expected: the DM approach has been under continuous development since the 1970s and is optimized for a wide variety of structures, whereas the potential of AI phasing is only beginning to be explored. With the aim of developing a more efficient phasing procedure than either method alone, we have integrated AI-PhaSeed with DM into a combined approach. The theoretical basis for using AI-derived phases to initiate DM phasing is outlined in Section 3.3. This protocol is denoted by DM+AI-PhaSeed. However, we have demonstrated that there are structures for which AI is not able to supply reliable phases, and these cases can be predicted with good accuracy by an RF model. Thus, we envisage that the combination of *AI-PhaseSeed* with DM could be conditioned by the results of the RF model prediction, by applying AI-PhaSeed only if the structure is classified as Class 1 by the RF model. In addition, by evaluating the efficiency of DM+AI-PhaSeed and DM separately for Class 0 and Class 1 structures (Fig. 9 ▸), we found that for structures with a data resolution (RES) of about 1.4 Å or worse (*i.e.* higher values such as 1.6 Å) DM+AI-PhaSeed outperforms DM for both classes. Thus, we can define an optimal solution strategy denoted by DM&AI-PhaSeed, according to which DM+AI-PhaSeed is applied to solve structures with lower data resolution (*i.e.* RES > 1.4 Å), while AI-PhaSeed alone is applied in cases of structures with higher resolution (*i.e.* RES ≤ 1.4 Å). From Fig. 10 ▸ it can be seen that this protocol has the highest efficiency on the entire structure dataset.

## 5. Discussion and perspectives

We have demonstrated that the method proposed by Carrozzini *et al.* (2025 ▸), when suitably implemented in the AI-PhaSeed procedure, can successfully phase crystal structures that cannot be solved by the currently available stand-alone AI PhAI neural network. When the AI-predicted phase seed is reliable, its performance approaches that achieved using true phase seeds.

The results were assessed in two steps. First, the reliability of the AI-generated phase seed was evaluated, monitored by the variables MPE_seed_ and CORR_seed_. Then, the phases obtained at the end of the AI-based solution process, where the AI-generated phases serve as a seed for traditional crystallographic methods, were assessed, using MPE_final_ and CORR_final_ as indicators. The performance of the first step was optimized using an RF classification model to predict the quality of the AI-generated phase seed. This model enabled us to analyse the dependence of AI performance on key variables related to the test structures, such as the percentage of the phase seed and the presence of heavy atoms.

For structures classified as optimal, the efficiency of AI-PhaSeed does not decline even at resolutions >1 Å, indicating that AI-PhaSeed is capable of phasing at resolution lower than the atomic level.

Besides the application of the method proposed by Carrozzini *et al.* (2025 ▸), we have made a step forward in integrating AI with *ab initio* phasing techniques, specifically DM, using the values of the phase seed generated by AI to support and reinforce the phase assignment performed by DM. To this end, we developed a DM+AI-PhaSeed integrated approach based on a modified tangent formula that combines AI-generated phases with those assigned by DM starting from random phases for reflections included in the phase seed.

The RF classification model also allowed us to optimize the application of the DM+AI-PhaSeed integrated approach. This was achieved by introducing a decision-making step based on structural features that are readily accessible after synthesis and X-ray measurement. With this fully integrated AI-based approach, we are able to phase real structures more efficiently than DM, even when the data resolution is >1 Å. This highlights the strong potential of the AI-PhaSeed method with low-resolution data.

In perspective, we expect that a substantial improvement in the AI-PhaSeed method and its integration with DM will result from the use of a neural network specifically trained on a larger number of investigated reflections. In the present study, we employed the PhAI neural network developed by Larsen *et al.* (2024 ▸), trained on reflection grids of fixed size (21 × 11 × 11). Our analysis identified that a critical factor for AI-PhaSeed performance is the percentage of the seed, defined as the number of seed reflections relative to the total number of experimental reflections.

Two key challenges must be addressed in the future to make this method suitable for routine structural determination of real structures: (i) extending AI-PhaSeed to structures with space groups other than *P*2_1_/*c*, including non-centrosymmetric structures, and (ii) developing alternative criteria for selecting the phase seed. Carrozzini *et al.* (2025 ▸) have laid the groundwork for addressing these challenges. In particular, the application to non-centrosymmetric structures could be addressed by applying a phase binning strategy, where continuous phase values in the range [0, 2π] are sampled by using two, three, four or six phase values equally distributed in the same range. Implementing these enhancements will require the design of a new neural network, offering opportunities for changes and improvements to its architecture.

## 6. Conclusions

The AI-based approach proposed by Carrozzini *et al.* (2025 ▸) has been implemented by integrating AI with traditional *ab initio* phasing techniques. Its first application, the AI-PhaSeed method, is presented here using the neural network originally trained by Larsen *et al.* (2024 ▸) on real structures of limited size and *P*2_1_/*c* symmetry.

We have demonstrated that the performance of the AI-PhaSeed method depends on two key factors: (i) the high extension of the phase seed, defined as the number of seed reflections relative to the total number of observed reflections, and (ii) the good quality of the phase values assigned to those seed reflections. When applied to structures matching both conditions, AI-PhaSeed can achieve optimal performance, in some cases surpassing that of classical DM. Notably, this advantage is observed even at data resolution lower than 1 Å, indicating that the AI approach has the ability to phase at resolution lower than the atomic level.

We have also developed machine learning tools, such as an RF classification model, to introduce advanced decision-making strategies in the phasing process. Guided by this model, we have developed a first-of-its-kind integration of AI-based tools with classical DM approaches, thereby enhancing the overall efficiency and reliability of crystal structure determination. This study highlights the strong potential of developing faster and more robust AI-based *ab initio* phasing methods, particularly for challenging cases involving low-resolution data, incomplete datasets or entirely novel protein folds.

Our results also suggest that the performance of AI-PhaSeed could be further improved by training a dedicated neural network specifically tailored/optimized to medium- and large-sized structures, incorporating the key features emphasized by the AI-PhaSeed approach. This development is planned for future investigations.

## Supplementary Material

Supporting information file. DOI: 10.1107/S1600576725008271/po5167sup1.pdf

## Figures and Tables

**Table 1:** Summary of key features of the test structures, presented as the variable names (first column), their description (second column) and the corresponding variability range (third column)

Variable name	Description	Variability range
*N*_asym_	Number of non-H atoms in the asymmetric unit	9.5–55
maxCellSize (Å)	Maximum linear dimension of the unit cell (maximum between *a*, *b*, *c*)	10.7–43.1
minCellSize (Å)	Minimum linear dimension of the unit cell (minimum between *a*, *b*, *c*)	3.68–14.8
Perc	Percentage of phase-seed reflections that meet the neural network’s input size criteria, over the total number of symmetry-independent reflections	5.03–53.9%†
Compl (%)	Crystallographic data completeness	29.7–100%
max*W*	Atomic weight of the heaviest element	∼12–238
Pseudo (%)	Pseudosymmetry percentage affecting atoms in the structure	0–96%
*N*_refl_	Number of measured symmetry-independent reflections	1508–20169
RES (Å)	Experimental data resolution	0.49–0.98
REFLEC_seed_	Number of reflections phased by AI	894–1042
RES_seed_ (Å)	Resolution for reflections phased by AI	0.59–1.37
Vol (Å^3^)	Volume of the unit cell	1005–3495

† On average, Perc exceeds 10%, ensuring sufficient data for effective phase prediction.

**Table 2:** Confusion matrix for the tenfold cross-validation RF model, showing predicted and actual class distributions Class labels were assigned using an optimal probability threshold, as determined by identifying the highest sensitivity value corresponding to a specificity of at least 80% (for correct classification of a significant portion of Class 1 structures) based on the global ROC curve [red dot in Fig. 5 ▸(*a*)]. Class 1 corresponds to MPE_seed_ ≤ Q1 and CORR_seed_ ≥ Q3, and Class 0 to MPE_seed_ > Q1 and CORR_seed_ < Q3.

	Actual: 0	Actual: 1
Predicted: 0	657	133
Predicted: 1	183	532

## Data Availability

The data supporting the results reported in this article, taken from the Crystallography Open Database, are available upon request from the authors.
