# Supplementary material for: The AI-based phase-seeding (AI-PhaSeed) method: early applications and statistical analysis
Source: J Appl Crystallogr. 2025 Oct 18;58(Pt 6):1859–69. doi: 10.1107/S1600576725008271 (PMC12810460; doi:10.1107/S1600576725008271)
Supplement: Supplementary file 1 [file j-58-01859-sup1.pdf]

Volume 58 (2025)

Supporting information for article:

The AI-based phase-seeding (*AI-PhaSeed*) method: early applications and statistical analysis

Benedetta Carrozzini, Francesca Fedele, Anna Moliterni, Liberato De Caro, Corrado Cuocci, Cinzia Giannini, Rocco Caliandro and Angela Altomare

The AI-based Phase-Seeding (AI-PhaSeed) Method: Early Applications and Statistical Analysis

Authors

Benedetta Carrozzini[a†], Francesca Fedele[a†], Anna Moliterni[a*], Liberato De Caro[a], Corrado Cuocci[a], Cinzia Giannini[a], Rocco Caliandro[a], Angela Altomare[a]

[a]Institute of Crystallography, National Research Council of Italy, via Amendola 122/o, Bari, 70126, Italy

Correspondence email: anna.moliterni@cnr.it

† These authors contributed equally to this work.

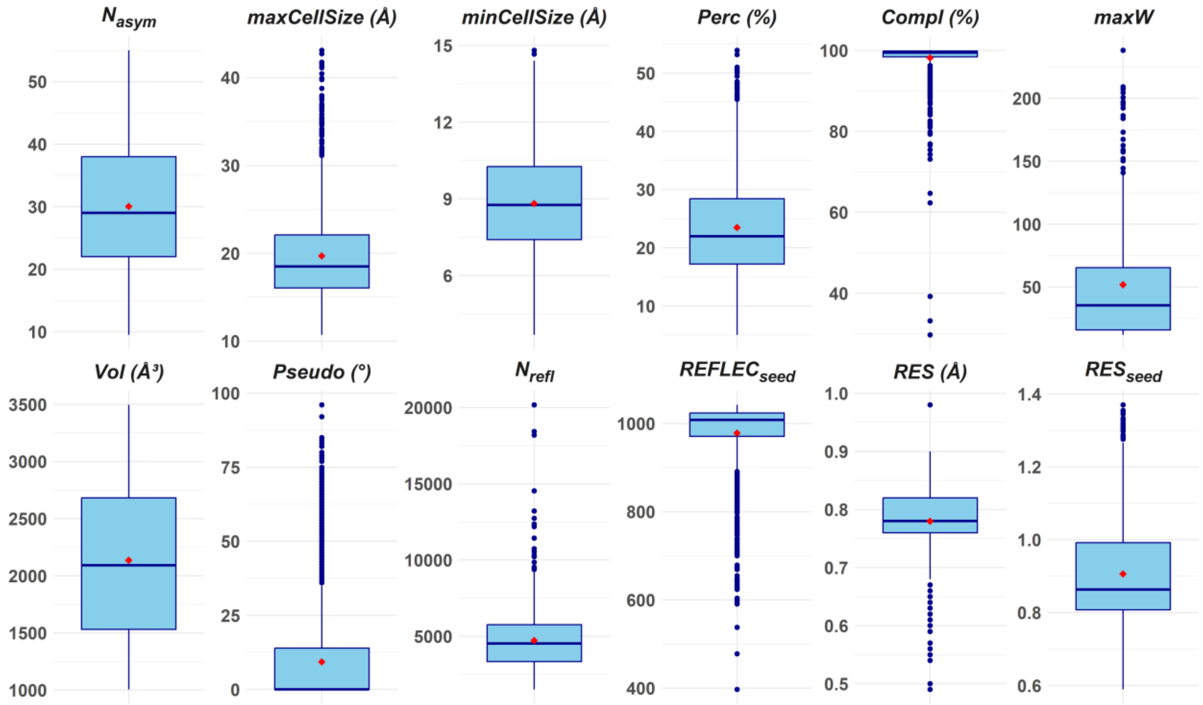

Figure S1 Boxplots of the test structure features, illustrating the distribution and variability of each feature. Red dots indicate the mean values.

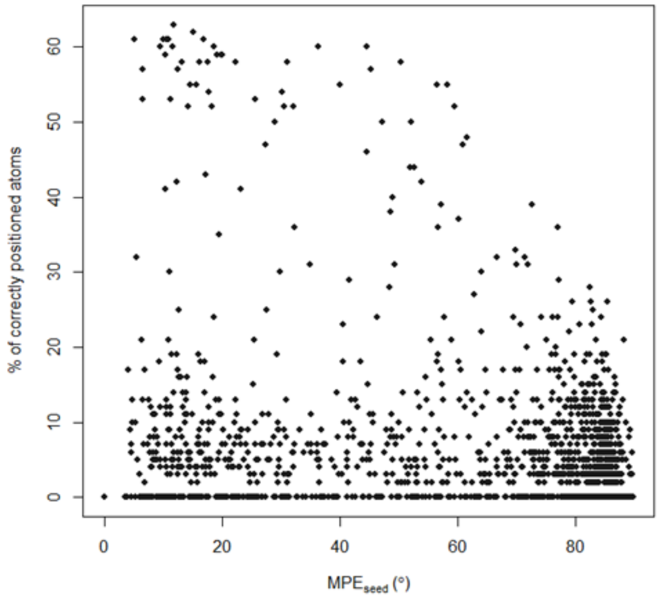

Figure S2 Scatter plot of the percentage of correctly positioned atoms in the electron density map calculated by using only the phase seed reflections *versus* the mean phase error calculated on the same reflections for all the test structures considered in this study. The phase values have been estimated by applying the PhAI CNN.

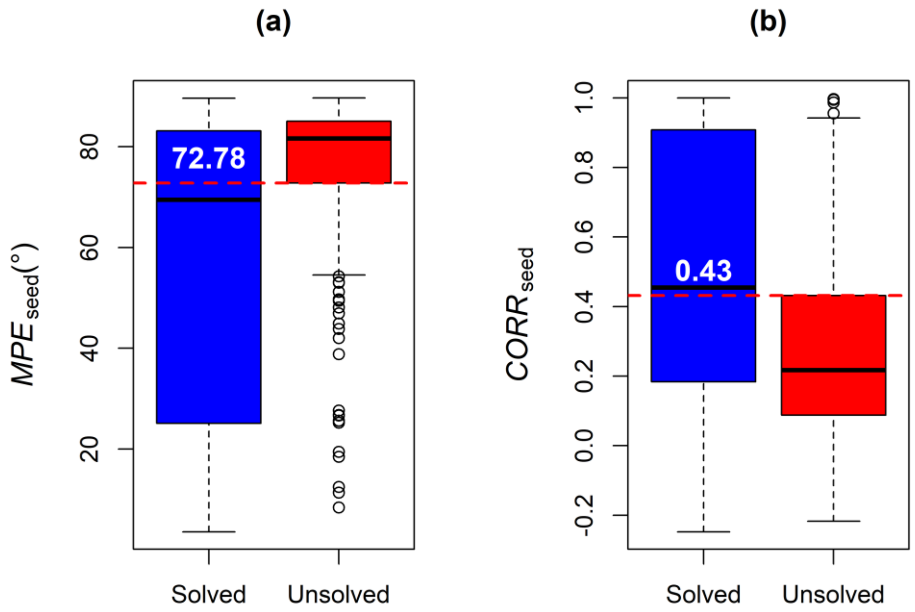

Figure S3 Comparison of MPE$_{seed}$ **(a)** and CORR$_{seed}$ **(b)** between solved and unsolved structures. The red dashed lines represent the first quartile for MPE$_{seed}$ and the third quartile for CORR$_{seed}$ both of the unsolved group. These reference values are reported on the corresponding boxplots.

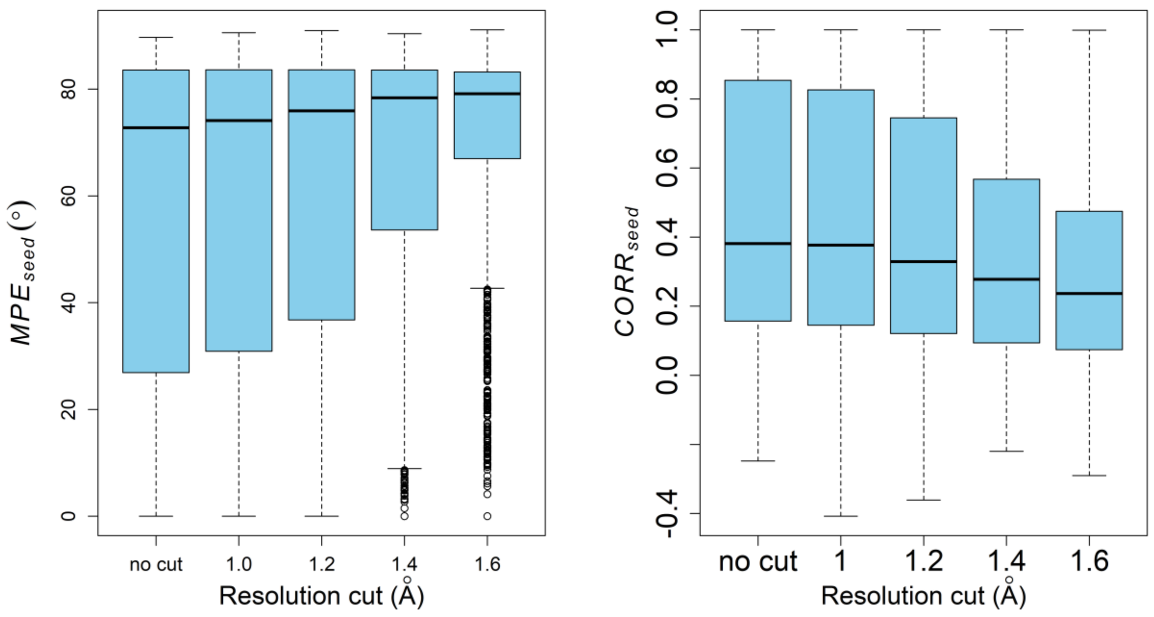

Figure S4 Boxplots of MPE_{seed} (left) and $CORR_{seed}$ (right) values for test structures at different input data resolution cut-offs ("no cut", 1 Å, 1.2 Å, 1.4 Å, 1.6 Å), highlighting the impact of resolution filtering on seeding accuracy.

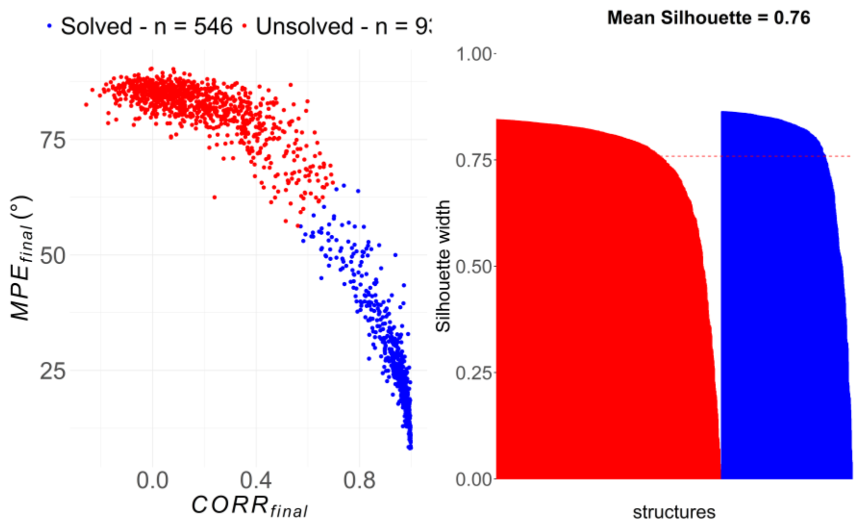

Figure S5 a) 2D scatter plot illustrating the k-means clustering with k=2 (Hartigan-Wong algorithm) results based on MPE_{seed} (y axis) and $CORR_{seed}$ (x axis) for data with a 1.6 Å resolution cut-off. Data points are color-coded to distinguish between "Solved structures" (blue) and "Unsolved structures" (red) with the legend indicating the number of observations in each category; **b)** Silhouette plot for k-means

clustering with k = 2. Each block contains a number of vertical bars equal to the number of elements assigned to that cluster (blue for solved and red for unsolved structures). The height of each bar represents the silhouette width, indicating how well the element fits its assigned cluster compared to the other one. Silhouette values closer to 1 correspond to better-defined clustering. The mean silhouette width reported above the plot quantifies the overall clustering quality; values greater than 0.7 are generally considered indicative of strong and well-separated clusters (Kaufman & Rousseeuw, 1990).

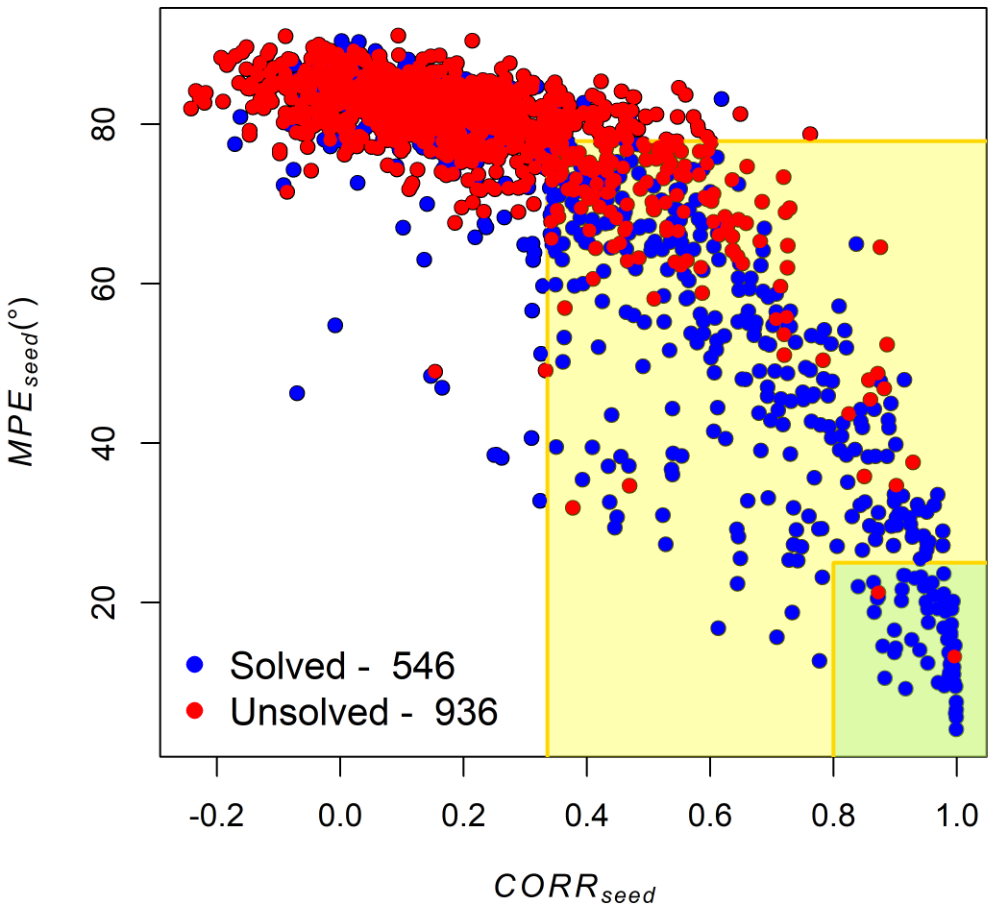

Figure S6 Scatter plot MPE_{seed} *vs* $CORR_{seed}$ for data with a 1.6 Å resolution cut-off showing clusters of solved (blue dots) and unsolved (red dots) structures. The green region corresponds to the area defined by $MPE_{seed} \leq 25°$ and $CORR_{seed} \geq 0.8$ where *AI* phases can be considered reliable, *i.e.*, sufficiently accurate to be treated as "true" phases. The yellow region corresponds to the area defined by the *RF classification model optimizing* values $MPE_{seed} \leq$ Q1 and $CORR_{seed} \geq$ Q3 and mostly contains solved structures, which accumulate at $CORR_{seed}$ values close to 1.

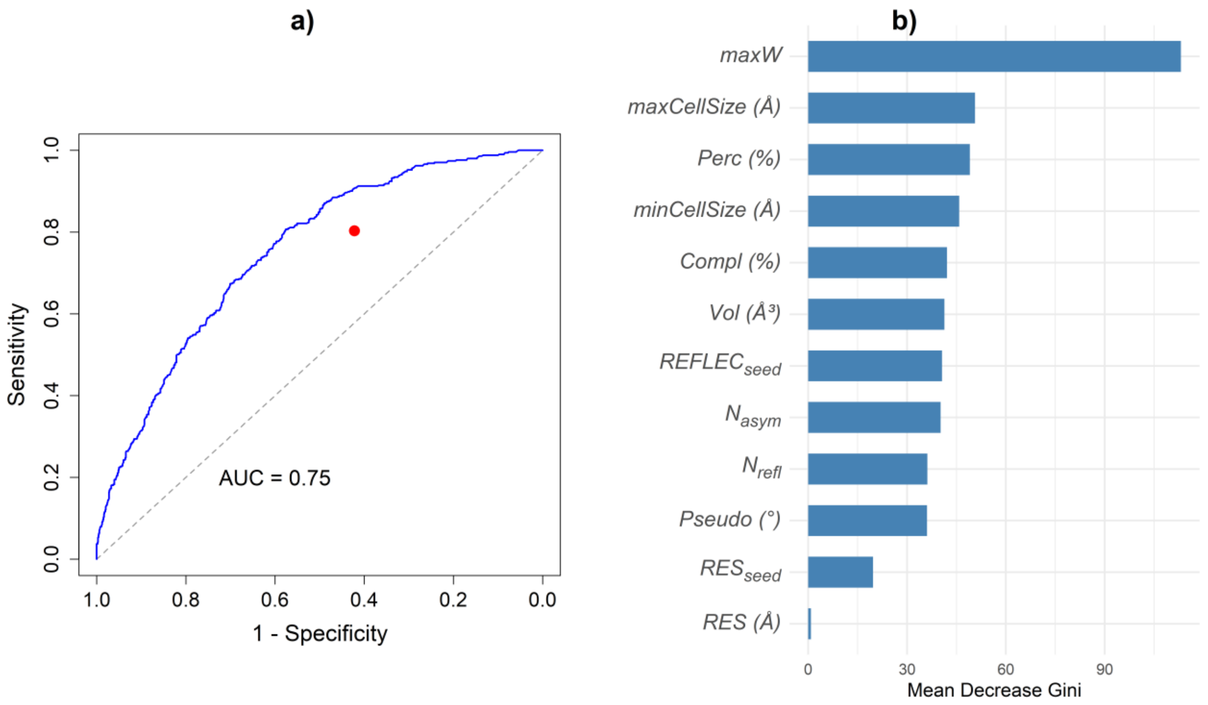

Figure S7 **a)** The ROC curve of the RF model for data with a 1.6 Å resolution cut-off illustrating sensitivity and specificity values for different thresholds. The red dot marks the optimal cut-off point, identified by the highest specificity value corresponding to a sensitivity at least of 80% (to correctly classify a significant portion of Class 1 structures). The AUC (Area Under the Curve) is reported as a measure of the overall performance of the model. **b)** Feature importance, ordered by the descending Mean Decrease Gini (Kaufman & Rousseeuw, 1990) resulting from the RF model.

Table S1 Confusion Matrix for 10-fold Cross-Validation RF model for data with a 1.6 Å resolution cut-off showing predicted and actual class distributions. Class labels were assigned using an optimal probability threshold as determined by identifying the highest sensitivity value corresponding to a specificity at least of 80% (to correctly classify a significant portion of Class 1 structures) from the global ROC curve (red dot in Figure S7a). Class 1 corresponds to $MPE_{seed} \leq$ Q1 and $CORR_{seed} \geq$ Q3; Class 0 to $MPE_{seed} >$ Q1 and $CORR_{seed} <$ Q3.

	Actual: 0	Actual: 1
Predicted: 0	566	99
Predicted: 1	413	404

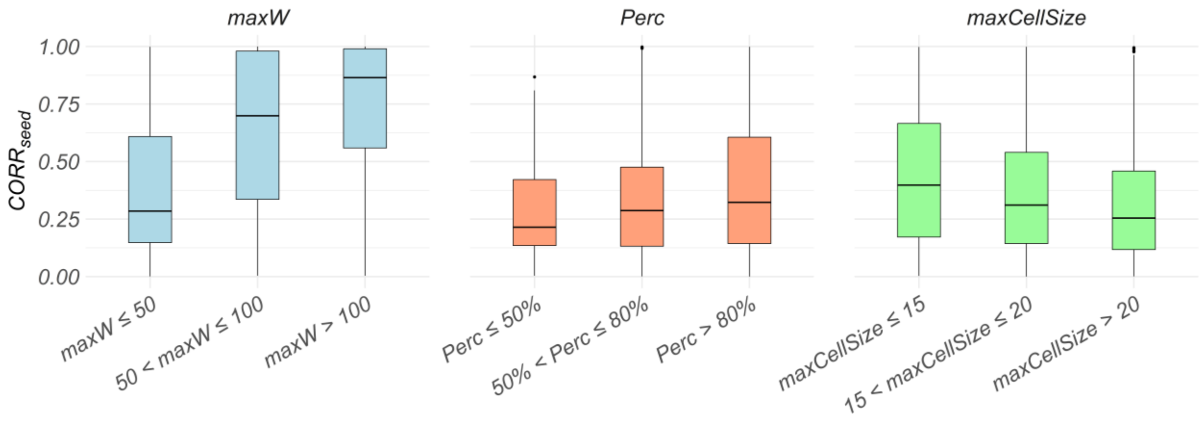

Figure S8 Distribution of $CORR_{seed}$ across different intervals of the three most important features according to the RF Mean Decrease Gini for data with a 1.6 Å resolution cut-off: maximum atomic number among the atomic elements ($maxW \leq 50$, $50 < maxW \leq 100$, and $maxW > 100$), percentage of reflections in the seed to total reflections ($Perc \leq 50\%$, $50\% < Perc \leq 80\%$, and $Perc > 80\%$) and maximum linear dimension of the unit cell (max between *a*, *b*, *c*) ($maxCellSize \leq 15$ Å, 15 Å< $maxCellSize \leq 20$ Å, and $maxCellSize > 20$ Å). The horizontal black bars indicate the median values.

References

Kaufman, L. & Rousseeuw, P. J. (1990). Finding Groups in Data: An Introduction to Cluster Analysis. Wiley.
